# Spatial organization of the tumor immune microenvironment in LAR+ triple-negative breast cancer

**DOI:** 10.3389/fimmu.2026.1810096

**Published:** 2026-05-22

**Authors:** Donatella Lucchetti, Alba Di Leone, Giulia Sabbatinelli, Federica Toma, Franco Antonio, Beatrice Cellini, Filomena Colella, Erica Pazzaglia, Chiara Parrillo, Luciano Giacó, Angela Santoro, Alessia Piermattei, Rita Colonna, Gianluca Franceschini, Alessandro Sgambato

**Affiliations:** 1 Multiplex Spatial Profiling Facility, Gemelli Science and Technology Park (GSTeP), Fondazione Policlinico Universitario ‘Agostino Gemelli’ IRCCS, Rome, Italy; 2 Department of Translational Medicine and Surgery, Università Cattolica del Sacro Cuore, Rome, Italy; 3 Fondazione Policlinico Universitario Agostino Gemelli IRCCS, Breast Unit, Department of Woman and Child Health and Public Health, Università Cattolica del Sacro Cuore, Rome, Italy; 4 Santoro Pathology Unit, Department of Woman and Child's Health and Public Health Sciences, Fondazione Policlinico Universitario Agostino Gemelli IRCCS, Rome, Italy; 5 Pathology Institute, Catholic University of Sacred Heart, Rome, Italy; 6 National Facility for Genomics, Human Technopole, Viale Rita Levi-Montalcini 1, Milan, Italy; 7 Breast Unit, Department Of Woman And Child’s Health And Public Health, Fondazione Policlinico Universitario A. Gemelli Irccs, Rome, Italy; 8 UOS Computational Biology and Bioinformatics, Gemelli Science and Technology Park (GSTeP), Fondazione Policlinico Universitario Agostino Gemelli IRCCS, Rome, Italy; 9 Pathology Unit, Department of Woman and Child’s Health and Public Health Sciences, Fondazione Policlinico Universitario Agostino Gemelli IRCCS, Rome, Italy

**Keywords:** Breast cancer, LAR, spatial proteomics, TNBC (triple negative breast cancer), tumor microenvironment

## Abstract

**Background:**

Triple-negative breast cancer (TNBC) is a heterogeneous disease lacking approved targeted therapies and standardized treatment regimens. Among its molecular subtypes, luminal androgen receptor-positive (LAR+) TNBC is characterized by reduced proliferative activity and a lower sensitivity to chemotherapy. The tumor immune microenvironment (TIME) plays a critical role in shaping treatment responses; however, its spatial organization and cellular composition in LAR+ TNBC remain poorly understood.

**Methods:**

In this exploratory study, we performed multiplex immunofluorescence analysis to characterize 18 immune and tumor cell subtypes in paired pre- and post-neoadjuvant therapy (NAT) samples from small, exploratory cohort of patients with LAR+ TNBC, stratified by pathological complete response (pCR). We assessed immune cell composition, expression of exhaustion markers, and spatial relationships among cellular populations to explore TIME features associated with different pathological responses.

**Results:**

Patients who achieved pCR displayed higher pre-treatment densities of specific immune subsets, including CD20^+^PD-1^+^, CD4^+^FOXP3^+^, and CD8^+^PD-1^+^TIM3^+^ cells, consistent with an immune-enriched microenvironment. Spatial analyses revealed distinct patterns between Responders (Resp) and Non-Responders (NoResp). In Resp, tumor cells (PANCK^+^) were initially located closer to PD-L1–expressing tumor cells (PANCK^+^PD-L1^+^), with this proximity decreasing after NAT. In contrast, in non-Resp, immunosuppressive tumor cells moved closer to tumor cells following treatment. Moreover, NAT in Resp was associated with a spatial repositioning of CD4^+^ and CD8^+^ T cells toward tumor cells. B cells and regulatory B cells (Bregs) also exhibited differential spatial dynamics between the two groups.

**Conclusions:**

This exploratory analysis describes distinct immune compositions and spatial arrangements of the TIME in LAR+ TNBC. Our findings suggest that specific immune enrichments and spatial remodeling patterns may differ between patients with different pathological outcomes, whereas the persistence of immunosuppressive niches characterizes non-Resp. Given the small sample size and the inclusion of immune checkpoint inhibitors in a subset of patients, all of whom achieved pCR, these observations should be considered strictly hypothesis-generating. Larger and more homogeneous cohorts will be required to validate these findings and to determine their potential clinical relevance.

## Introduction

Breast cancer is the most common cancer affecting women worldwide, accounting for 31% of all cancers in women. Currently, the breast cancer survival rate has improved but breast cancer remains a leading cause of death in women aged 30–60 years globally (1). Breast cancer is a highly heterogeneous disease, and from a molecular perspective, they can be classified into 5 subtypes according to gene expression profiles: luminal A, luminal B, human epidermal receptor 2 (HER2)-enriched, triple-negative breast cancer (TNBC) and claudin-low (2). This molecular diversity represents one of the main challenges in clinical management and in the development of targeted therapies.

TNBC is characterized by a lack of expression of estrogen (ERs), progesterone (PgRs), and HER2 receptors. This subtype accounts for approximately 15–20% of all breast cancers but is associated with a high mortality rate because of its aggressive nature (3). Recent studies have further subdivided TNBC into categories on the basis of gene expression profiles, with Lehmann’s molecular classification identifying subtypes such as basal-like (BL1 and BL2), mesenchymal, mesenchymal-stem-like, immunomodulatory, and luminal androgen receptor (LAR) (4). Androgen receptor (AR) positivity is observed in 12–50% of TNBC (5–7), generating interest in AR-expressing TNBC subtypes as potential targets for novel therapies (8, 9).

Although patients with early-stage TNBC are often treated with adjuvant chemotherapy, they continue to have poorer prognoses, with high rates of distant metastasis (10). Given the limited availability of effective therapies, because the lack of specific molecular targets represents a major unmet clinical need, chemotherapy remains the main treatment option in neoadjuvant, adjuvant, and metastatic settings, with approximately 30–40% of patients achieving a complete pathological response (pCR) after neoadjuvant chemotherapy, significantly improving survival (11, 12). However, patients with residual disease after neoadjuvant therapy have a sixfold increased risk of recurrence and a twelvefold increased risk of death from metastatic disease (12–14).

Compared with basal TNBC subtypes, LAR tumors are generally less proliferative and have a lower sensitivity to chemotherapy (15). Studies have shown that AR expression is associated with a lower histological grade and clinical stage (16), whereas the absence of AR is correlated with a greater risk of recurrence and metastasis (17). However, the prognostic significance of AR remains controversial, with some studies linking AR expression to improved disease-free survival, whereas others have reported no significant impact (18). Understanding the role of AR across different molecular contexts is crucial for developing effective targeted therapies (19).

An emerging area of TNBC research is the study of the tumor immune microenvironment (TIME), which plays a crucial role in modulating tumor progression and the response to therapy. TIME comprises immune cells, stromal cells, and extracellular matrix components that interact in complex ways with tumor cells, influencing disease behavior. Recent studies have highlighted how different TNBC subtypes may exhibit distinct immune tumor microenvironments, which may influence treatment response and clinical outcomes (20). Tompson and colleagues reported that non-LAR patients are twice as likely to respond to neoadjuvant therapy (NAT). Moreover, non-LAR TNBCs exhibit elevated immune-related gene expression, which is strongly associated with the therapeutic response (pCR) (21). Despite increasing evidence supporting a role of the tumor immune microenvironment (TIME) in shaping treatment response in TNBC, the spatial organization and cellular composition of immune niches in LAR+ tumors remain largely unexplored. Most available studies rely on bulk transcriptomic approaches, which fail to capture the spatial context and cell–cell interactions that critically regulate immune function. Moreover, the rarity of the LAR+ subtype and the limited availability of paired pre- and post-treatment samples have hindered detailed spatial investigations in this setting. In this exploratory study, we employed multiplex immunofluorescence and spatial analysis to characterize the immune composition and spatial organization of the TIME in a small cohort of LAR+ TNBC patients undergoing neoadjuvant therapy. By comparing patients who achieved pathological complete response (pCR) with those who did not, we aimed to generate hypotheses regarding immune-tumor spatial interactions associated with different pathological outcomes, rather than to establish definitive predictive biomarkers. Our results provide a spatially resolved view of the TIME in this understudied TNBC subtype and offer a conceptual framework for future validation studies in larger and more homogeneous cohorts.

## Materials and methods

### Patients and tissue samples

Formalin-fixed, paraffin-embedded (FFPE) tumor samples from twelve LAR breast cancer (BC) patients who underwent neoadjuvant therapy (NAT) between 2016 and 2024 at Fondazione Policlinico Universitario Agostino Gemelli IRCCS in Rome were retrieved from the archive of the Pathology Department. The diagnoses were confirmed by an expert pathologist (Angela Santoro) according to the 2019 WHO classification of breast cancer (22).

Among the twelve patients, four achieved pathological complete response (pCR). Notably, neoadjuvant treatment regimens were heterogeneous within the Resp group, with three out of four patients receiving Pembrolizumab in combination with chemotherapy, while one patient received chemotherapy alone. All patients who received Pembrolizumab achieved pCR. Clinical and pathological data were collected from medical records. Hormone receptor (HR) and HER2 status were assessed in tumor tissue from both the primary core needle biopsy performed before neoadjuvant therapy and the posttherapy tissue samples. AR testing was conducted for all patients. The following clinicopathological information was retrieved from medical records and is described in **Table 1**: (1) age at diagnosis; (2) TNM stage according to the American Joint Committee on Cancer (AJCC) Cancer Staging Manual, 8th Edition (23); (3) histological grade of the primary tumor; (4) pathological histotype (including ductal and other subtypes); and (5) HER2, Ki67, and AR status of the primary tumor. HER2 gene status was evaluated by fluorescence (FISH) for 2+ cases via immunohistochemistry. Patient characteristics are summarized in **Table 1**.

**Table 1:** Patients clinicopathological information.

Id Patient	Age at diagnosis	TNM stage	Treatment	Pre-NACT AR+ (%)	Pre-NACT Ki67%	Pre-NACT HER2 state	Mandard class	pCR
1	30	cT2cNo	1	6	70	0	TRG1	yes
2	49	cT2cN1	1	95	25	0	TRG4	no
3	57	cT2cN2	1	2	60	2+*	TRG2	no
4	60	cT2cN2	2	80	50	2+*	TRG4	no
5	60	cT2cN1	1	5	10	0	TRG3	no
6	74	cT2cN1	2	90	60	2+*	TRG4	no
7	44	cT1cN2	2	90	60	2+*	TRG4	no
8	68	cT2cN0	2	100	25	2+*	TRG4	no
9	71	cT2cN1	2	5	50	0	TRG3	no
10	31	cT2cN0	3	50	70	0	TRG1	yes
11	57	cT2cN0	4	25	70	0	TRG1	yes
12	59	cT2cN1	3	90	80	1+	TRG1	yes

* HER2 gene status was evaluated by fluorescence (FISH) for immunohistochemistry 2+ cases.

1 EC + CBDA + Taxolo.

2 EC + Taxolo.

3 EC+Taxolo + Pembrolizumab.

4 EC+CBDA+ Taxolo+ Pembrolizumab.

### Marker evaluation

Tumor ER, PR, HER2, Ki67, and AR status was assessed by immunohistochemical staining and recorded in the pathology report. Immunostaining for ER, PR, and Ki67 was evaluated according to ASCO guidelines (24) while HER2 was assessed by immunostaining and/or *in situ* hybridization. ER and PR were considered positive if ≥1% of cells were stained. HER2 was scored by immunostaining as recommended, from 0 to 3 +. HER2 gene status was evaluated by fluorescence (FISH) for immunohistochemistry in 2+ cases.

### Multiplex immunofluorescence staining

The eight-color multiplex immunolabeling was performed via Opal chemistry (Opal 7-color IHC kit, PerkinElmer, Waltham, USA, Cat. No. NEL87100KT) containing the following TSA fluorophores: Opal 480, Opal 520, Opal 570, Opal 620, Opal 690, Opal 780 and spectral DAPI. Opal 540 and Opal 650 were also used to create a panel of 8 markers to characterize the subpopulations of tumor-infiltrating immune cells. The panel included primary antibodies against CD8 (clone 144B, Abcam), CD4 (clone EP204, Cell Signaling), CD20 (clone L26, Cell Signaling), FOXP3 (clone 23EA/E7, Abcam), TIM3 (clone D5D5R, Cell Signaling), PD-1 (clone D4W2S, Cell Signaling), PD-L1 (clone E1L3N(R), Cell Signaling), and pan-CK (clone AE1/AE3, Cell Signaling) antibodies. CD20+PD-1+ cells identified as Bregs (PD-1+ immunoregulatory Bcells) (25), PANCK+FOXP3+ as FOXP3-expressing tumor cells with pro-tumor activity (26) and CD8+PD-1+TIM3+ as exhausted T cell (27). Detailed information on the antibodies, including their dilutions and retrieval buffers, is given in **Table 2**. We used a Leica BOND RX autostainer for automated staining, and all fluorophores and DAPI were prepared according to the manufacturer’s guidelines.

**Table 2:** Opal 8-plex antibody panel.

Order immune panel	Antibody	Supplier	Clone	Diluition factor	Opal pairing	Retrival and incubation time
1	FOXP3	Abcam	23EA/E7	1:150	570	ER2 30’
2	TIM-3	CST	D5D5R	1:100	540	ER2 30’
3	CD4	CST	EP204	1:100	690	ER2 30’
4	PD-1	CST	D4W2J	1:200	620	ER2 30’
5	CD8	Abcam	144B	1:100	480	ER2 30’
6	CD20	CST	L26	1:3000	650	ER2 30’
7	PD-L1	CST	E1L3N(R)	1:400	520	ER2 30’
8	Pan-CK	CST	AE1/AE3	1:100	780	ER2 45’

### Multispectral imaging and bioinformatics analysis

Multiplex-stained slides were imaged via the Vectra Polaris™ Automated Quantitative Pathology Imaging System (Akoya Biosciences) at 20X magnification. For each sample, images were analyzed via whole slide imaging (WSI). InForm Image Analysis software (version 3.0.0, Akoya Biosciences) was used to unmix multispectral images via a spectral library built from single fluorophore-stained control tissues containing fluorophores with defined spectral emission peaks. A selection of representative multispectral images was used to train the inForm software to generate algorithms. Tumor tissue was segmented on the basis of the identification of cells positive for the pan-cytokeratin antibody, allowing differentiation between infiltrating immune cells within the tumor area and those in the surrounding stroma. Single-cell segmentation was performed via nuclear counterstaining. Cell phenotyping was based on the detection of colocalized cell surface or intracellular markers, and algorithms were generated and applied in the batch analysis of all acquired multispectral images. Data preprocessing and analysis were performed via ALOA pipeline (28). Specifically, population counts were normalized by calculating the ratio between positive cells for a specific marker and each sample’s total cell count, then scaled by the average total cell count of the respective group. Distances were measured using nearest-neighbor analysis and standardized via Z-score. To account for multiple testing in the spatial distance analyses, p-values were adjusted using the Bonferroni correction. A p-value < 0.05 was considered statistically significant.

## Results

### Patient characteristics and immune panel description

**Figure 1:**
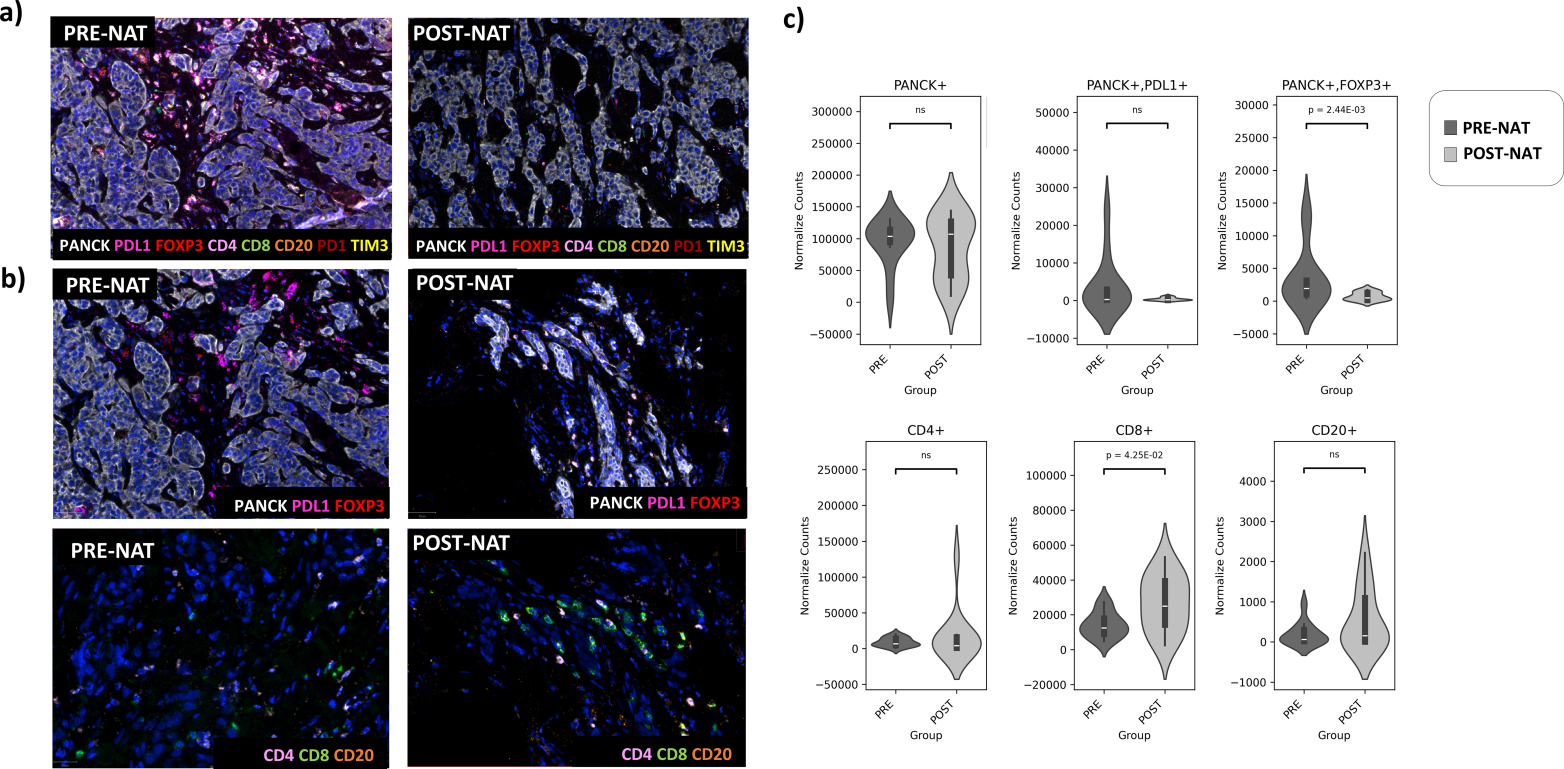
**(a)** Representative nine-color multispectral images of stained mIF panels of LAR+ TNBC sample before (*left panel*) and after (*right panel*) NAT-. Original magnification ×20. Immune markers and color codes are indicated in the legend. **(b)** Representative multispectral images of a LAR+ TNBC sample before (*left panels*) and after (*right panels*) NAT in Resp patients stained with PD-L1, PANCK and FOXP3 markers. **(c)** Densities of immune infiltrates and tumor cells (CD4^+^, CD8^+^, CD20^+^, PANCK^+^) in pre- and post-NAT samples. Data are shown as normalized counts.

We evaluated differences in cell population counts and distances by comparing sections before and after NAT in both the group that achieved pCR (Resp) (n=4) and the group that did not achieve pCR (NoResp) (n=8). Additionally, we compared pre-NAT and post-NAT sections between the Resp group and the NoResp group. To evaluate TIME diversity in the samples before and after NAT in LAR+ TNBC patients, we measured the 18-cell subtype composition across the combination of eight diversity markers described above (**Table 3**). The mIF panel was designed to identify T cells (CD4+ and CD8+ cells), B cells (CD20+ cells), T regulatory cells (CD4+FOXP3+ cells), tumor cells (pan-CK+ cells), and immune checkpoint molecules such as PD-1 and TIM-3 on immune cells, as well as PD-L1 and FOXP3 on tumor cells (**Figure 1a**).

**Table 3:** Co-expression markers and associated phenotypes.

Marker co-expression	Phenotype
PancK+	Tumor cells
Panck+FoxP3+	FoxP3 Immunosuppressive tumor cells (1)
Panck+PDL1+	PDL1+ Immunosuppressive tumor cells (2)
CD20+	B cells
CD20+PD1+	Breg (3)
CD8+	CD8+ T cells (4)
CD8+FoxP3+	CD8+ Treg (4)
CD8+PD1+	CD8+ exhausted (4)
CD8+TIM3+	CD8+TIM3+ exhausted (4)
CD8+TIM3+PD1+	CD8+TIM3+PD1+ exhausted (4)
CD8+PD1+FoxP3+TIM3+	CD8+ Treg highly exhausted (4)
CD4+	CD4+ T cells (4)
CD4+FoxP3+	CD4+ Treg (4)
CD4+PD1+	CD4+ exhausted (4)
CD4+TIM3+	CD4+TIM3 exhausted (4)
CD4+PD1+FoxP3+	CD4+PD1+ Treg exhausted (4)
CD4+PD1+TIM3+	CD4, TIM3 exhausted (4)
CD4+PD1+FoxP3+TIM3+	CD4 Treg highly exhausted (4)

### Immune composition of the TIME in LAR+ TNBC before and after neoadjuvant therapy

**Figure 2:**
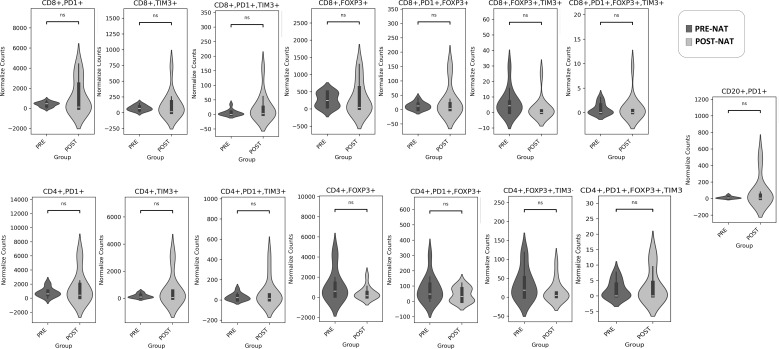
Detailed characterization of the immune microenvironment before and after NAT. The densities of exhausted T cells, Tregs and Bregs are presented as normalized counts. Statistically significant differences in normalized counts were observed in CD8^+^PD-1^+^TIM3^+^, CD8^+^FOXP3^+^, and CD20^+^PD-1^+^ cells.

Specifically, we observed a reduction in PANCK^+^FOXP3^+^ tumor cells, which may contribute to immune evasion and pro-tumor activity, accompanied by an increase in tumor-infiltrating lymphocytes (TILs), particularly CD8^+^ T cells, following NAT. (**Figures 1b, c****).** No significant differences were found in the exhausted T-cell population or Bregs following NAT (**Figure 2****).**

### Distinct immune phenotypes characterize patients with different pathological outcomes

**Figure 3:**
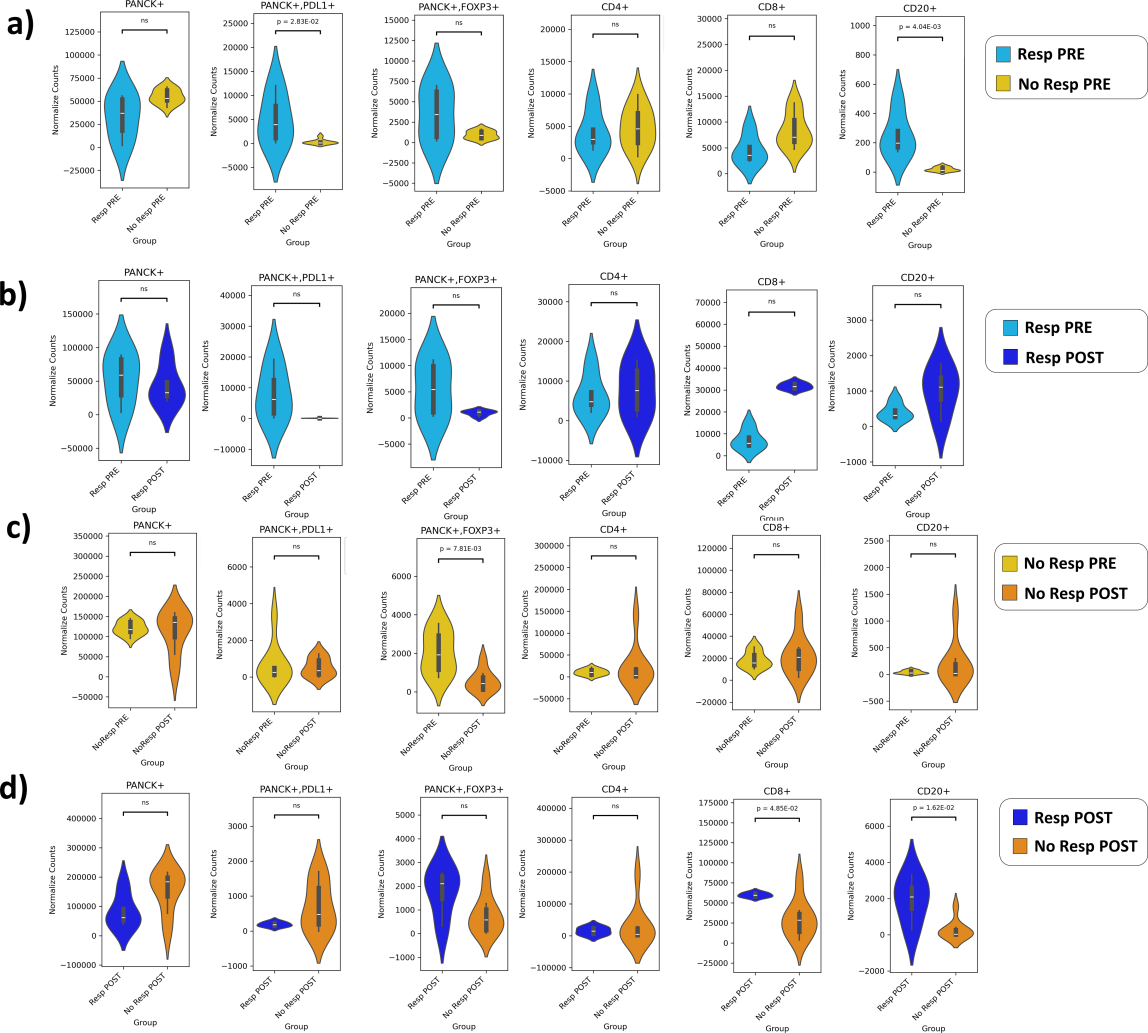
Normalized counts of TILs (CD4^+^, CD8^+^, and CD20^+^) and tumor cell populations (PANCK^+^, PANCK^+^PD-L1^+^, and PANCK^+^FOXP3^+^) across all study groups. **(a)** Resp PRE vs NoResp PRE; **(b)** Resp PRE vs Resp POST; **(c)** NoResp PRE vs NoResp POST; **(d)** Resp POST vs NoResp POST.

A comparison of immune phenotypes between the Resp and NoResp groups prior to NAT revealed significant differences in several cell populations. Importantly, in this cohort, all patients who received immune checkpoint inhibitors achieved pCR, suggesting a potential association between treatment regimen and response status, which should be taken into account when interpreting these TIME differences. Within this context, the Resp group presented a greater CD20+ cell density before NAT than the NoResp group did (**Figure 3a**). Moreover, immunosuppressive tumor cells were more abundant in the Resp group prior to NAT than in the NoResp group (PANCK+PD-L1+) (**Figure 3a**). Compared with that in the NoResp group, the TIL density in the Resp group after NAT remained unchanged, whereas immunosuppressive tumor cells were almost absent, as expected (**Figure 3b**). Notably, the frequency of PANCK^+^FOXP3^+^ cells did not change across conditions in Resp, showing no significant differences between pre- and post-NAT, while in Non-Resp a significant alteration was detected (**Figures 3b, d**). Conversely, CD8, and CD20 cells were increased in post-NAT Resp compared with post-NAT NoResp group (**Figure 3d**).

### Immune cell subtypes differ between patients with different pathological outcomes

**Figure 4:**
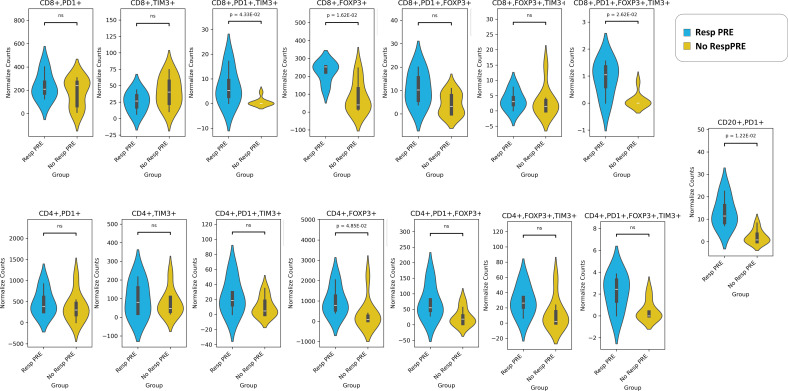
Detailed characterization of the immune microenvironment in Resp and NoResp before NAT. Significant differences in the abundances of exhausted T cells (CD8^+^PD-1^+^FOXP3^+^TIM3^+^; CD8^+^PD-1^+^TIM3^+^), regulatory T cells (CD4^+^FOXP3^+^), and regulatory B cells (CD20^+^PD-1^+^) were evident between the groups. The data are shown as normalized counts.

**Figure 5:**
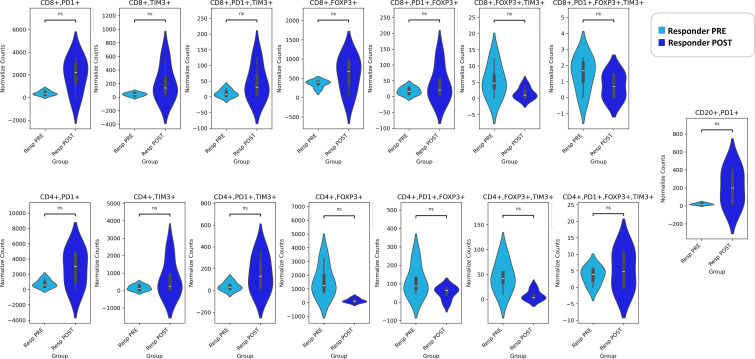
Detailed characterization of the immune microenvironment in Resp patients before and after NAT. The data are presented as normalized counts.

**Figure 6:**
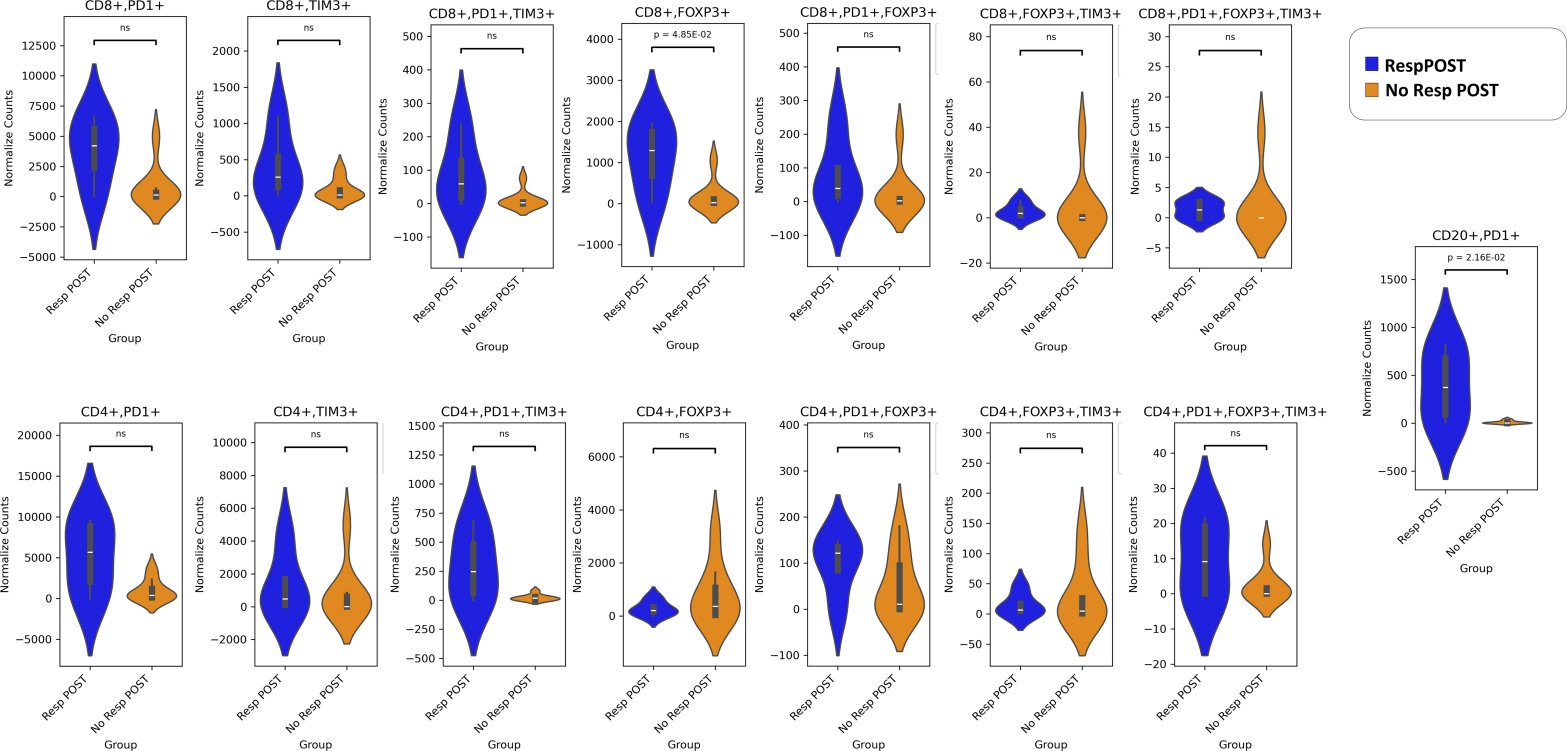
Detailed characterization of the immune microenvironment in NoResp patients and Resp after NAT. The data are presented as normalized counts.

**Figure 7:**
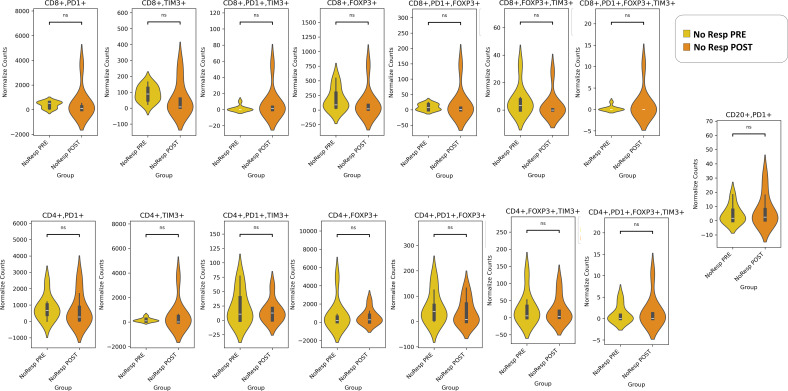
Detailed characterization of the immune microenvironment in NoResp patients before and after NAT. The data are presented as normalized counts.

In our panel, we evaluated the exhaustion of T cells by the PD-1, TIM3, and FOXP3 markers in both CD4+ and CD8+ T cells. Regulatory T cells (Tregs) are identified by FOXP3 expression, whereas regulatory B cells (Bregs) are characterized by PD-1 expression (25). Our results revealed that both Tregs (CD4+FOXP3+ and CD8+FOXP3+) and Bregs (CD20+PD-1+) were more numerous in the pre-NAT Resp than the NoResp group (**Figure 4**). We also noted that the density of exhausted CD8+PD-1+FOXP3+TIM3+ immune cells (27) was greater in the Resp group than in the NoResp group before NAT (**Figure 4**). Furthermore, NAT administration did not affect the immune subtypes in the Resp group (**Figure 5****).** Resp showed a significant post-NAT increase in CD8^+^FOXP3^+^ T cells and CD20^+^PD-1^+^ B cells (**Figure 6****),** whereas in the NoResp group NAT resulted in no change in exhausted immune cells (**Figure 7****).**

### Distinct spatial patterns identified by neighborhood analysis

**Figure 8:**
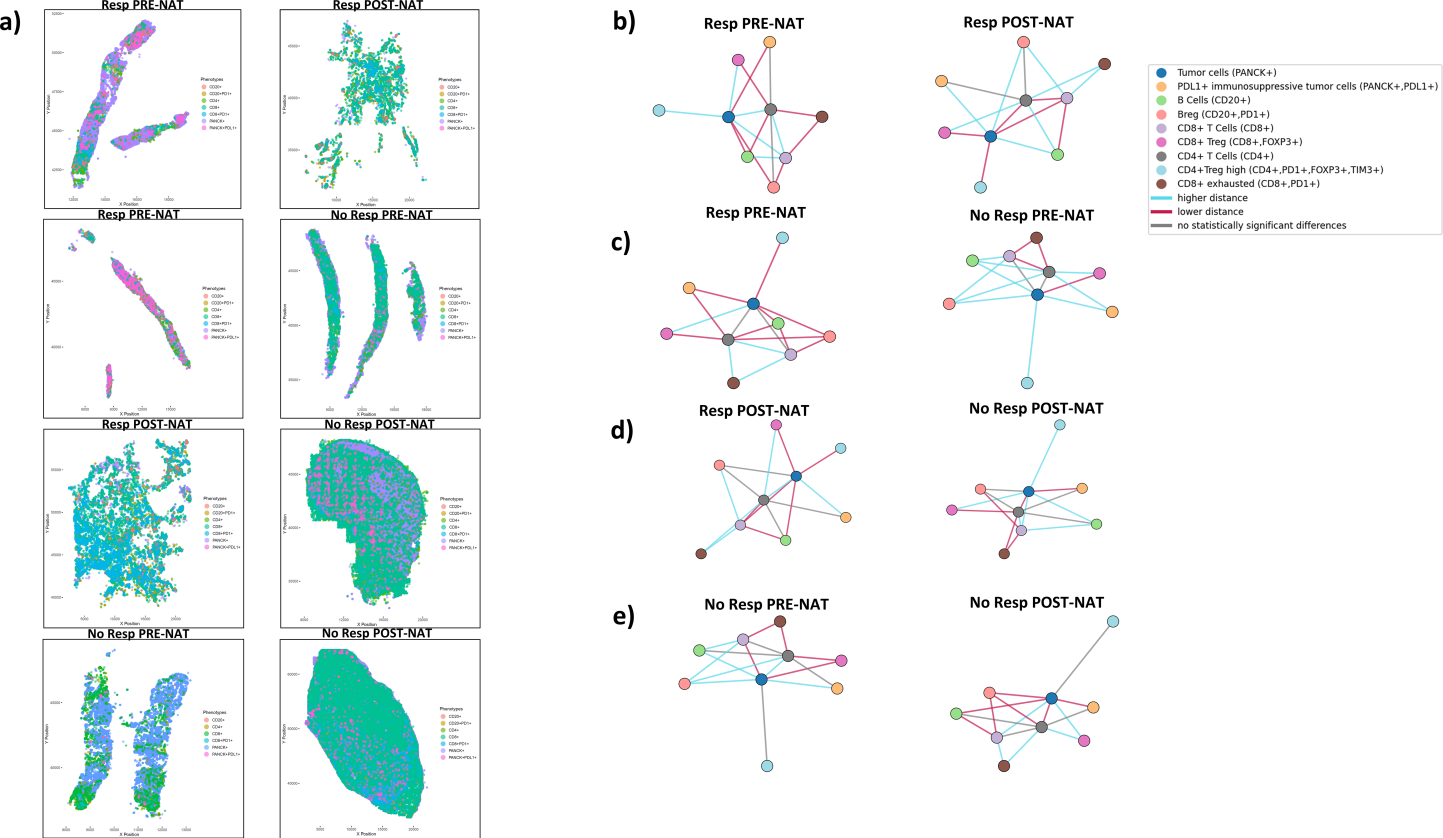
**(a)** Representative map plot images generated by nearest neighbor analysis, illustrating the distance between the closest neighboring cells**. (b–e)** Schematic summary of the results obtained from distance analyses between different cell populations. Blue indicates increased distance; red indicates decreased distance; gray indicates no statistically significant change.

Neighborhood analysis of different cell populations revealed a complex network of interactions within the pretreatment TIMEs (**Figure 8a**). Distance analysis revealed that tumor cells (PANCK+) were closer to PD-L1-expressing tumor cells (PANCK+PD-L1+) in the Resp group than in the NoResp group (**Figure 8c**). Following NAT, an increased distance between PANCK+ and PANCK+PD-L1+ cells was observed in the Resp group (**Figure 8b**). Notably, when comparing Resp and NoResp groups after NAT, PANCK+ and PANCK+PD-L1+ cells were positioned at a greater distance in the Resp group, whereas in the NoResp group these populations remained in closer proximity (**Figure 8d**). Notably, NAT in the NoResp group affected PD-L1-expressing tumor cells (PANCK+PD-L1+) that moved closer to the tumor cells (PANCK+) (**Figure 8e**). Compared with those in the Resp group, CD8+ exhausted T cells in NoResp pre-NAT were located closer to CD8+ T cells (**Figure 8c**). In Resp patients, NAT administration resulted in a reduction in the distance between T cells and PANCK+ cells (**Figure 8b**). In the NoResp group, the therapy promoted the exclusion of exhausted CD8+ T cells from both the CD4+ and CD8+ T-cell compartment, with a greater spatial separation from the PANCK+ population observed for CD8+ and CD8+ Treg. (**Figure 8e**). In addition, Breg and B cells were initially closer to PANCK+ cells in the Resp group, and NAT led to their displacement (**Figure 8b**). Otherwise, in NoResp patients, Bregs and B cells were initially positioned farther from tumor cells before NAT, but the therapy promoted their closer association with tumor cells (**Figure 8e**).

## Discussion

This study describes differences in immune cell populations and spatial arrangements within the tumor microenvironment of LAR+ TNBC patients with different pathological outcomes.

Currently, one significant challenge in cancer treatment is T-cell exhaustion within TIME, which plays a pivotal role in tumor progression. In the context of cancer, T cells engage in a continuous response to antigenic stimulation, gradually losing their functionality and entering a state known as “functional exhaustion”. This condition is characterized by the sustained upregulation of inhibitory receptors, such as programmed cell death protein-1 (PD-1), lymphocyte-activation gene-3 (LAG-3), and T-cell immunoglobulin and mucin domain-containing protein-3 (TIM-3) (29).

In this study, we used a multiplex immunofluorescence analysis to analyze 18 subtypes of immune and tumor cells in paired pre- and post-NAT samples from patients with LAR+ TNBC stratified by pathological complete response (pCR). Notably, the Resp group displayed higher pre-NAT levels of specific immune phenotypes, including CD20+PD-1+, CD4+FOXP3+, and CD8+PD-1+TIM3+ cells, suggesting that an enriched immune environment may be associated with a favorable response to treatment. The presence of immune checkpoint markers such as PD-1 and TIM-3 on these cells further points to an actively regulated immune landscape within Resp patients, indicating that these cells may play a central role in facilitating treatment efficacy (30). These findings are consistent with a cohort of TNBC RNA-Seq data investigated by Thompson and colleagues that revealed a correlation between a lower level of infiltrating T cells and a reduced pCR rate after NAT (21).

Our spatial analyses further revealed distinct immune interactions, and one of the most striking findings was the altered proximity between tumor cells and immune populations, particularly in the Resp and NoResp groups. In the Resp group, tumor cells (PANCK+) were found to be in closer proximity to immunosuppressive tumor cells (PANCK+PD-L1+) prior to treatment. However, post-NAT, this proximity decreased, suggesting that the treatment may have led to a reduction in immunosuppressive forces within the tumor. This shift in the immune microenvironment could contribute to the improved response observed in these patients. Conversely, in the NoResp group, immunosuppressive tumor cells (PANCK+PD-L1+) moved closer to the tumor cells after NAT, indicating that therapy may not have effectively altered the immunosuppressive network within the tumor, which might explain the lack of response to therapy.

We speculate that immune-suppressive tumor cells could be responsible for maintaining the distance between CD8+ T cells and cancer cells in the NoResp group after NAT, in addition to the expulsion of exhausted CD8+ T cells as a direct effect of therapy. This persistent immune suppression could prevent the immune system from developing an effective response against the tumor.

Another key observation concerned the spatial relationship between T cells and tumor cells. In the Resp group, both CD4^+^ and CD8^+^ T cells were located further from tumor cells (PANCK^+^) prior to NAT, and following treatment their proximity to tumor cells increased, suggesting that NAT may facilitate the repositioning of effector T cells toward the tumor, potentially enhancing their ability to target and eliminate cancer cells. In contrast, in the NoResp group, CD8^+^ T cells were already in close proximity to tumor cells prior to NAT, yet this association was lost following treatment, while CD4^+^ T cells showed no significant spatial relationship with tumor cells at either timepoint. This pattern suggests that in NoResp tumors, therapy may fail to sustain or promote effective T cell–tumor cell interactions, potentially reflecting a less permissive immune microenvironment.

Furthermore, our analysis of Breg and B-cell positioning revealed significant differences between the two groups of patients. In the Resp group, B cells and regulatory B cells (Breg) were initially closer to tumor cells, but NAT displaced them, suggesting that therapy might alter the spatial organization of immune cells in a way that promotes a more favorable immune environment. In contrast, in the NoResp group, B cells and Bregs were positioned farther from tumor cells before NAT, but therapy brought them closer to the tumor, potentially indicating that immune cells were recruited to the tumor site but did not achieve an effective response. This shift might further explain the lack of therapeutic success in these patients, as it suggests that while immune cells are present, their ability to interact with and eliminate tumor cells is limited. Classical studies have highlighted the molecular interactions between PD-1 and PD-L1 in the identification of novel immunotherapies (31). Recent studies have suggested that, beyond its role in enabling tumor cells to evade immune surveillance, PD-L1 also functions as a key effector molecule that is involved in the activation of intrinsic signaling pathways in tumor cells, preventing apoptosis or promoting tumor progression and growth in an immune-independent manner (31). For this reason, further molecular investigations of the PD-L1+ immunosuppressive tumor cell population are essential to better understand its complex role in LAR+ TNBC patients who do not respond to NAT. This study has several important limitations that should be carefully considered when interpreting the results. First, the cohort size is small, reflecting the relative rarity of the LAR+ TNBC subtype and the limited availability of paired pre- and post-treatment specimens. As a consequence, the number of patients achieving pCR was low, preventing robust statistical modeling and precluding any definitive conclusions regarding the predictive value of the observed immune and spatial features.

Second, the heterogeneity of neoadjuvant treatment regimens represents a relevant confounding factor. In particular, a subset of patients received immune checkpoint inhibitors, and all of these patients achieved pCR. This introduces a strong bias that limits the attribution of the observed TIME patterns specifically to therapy response and prevents disentangling the effects of cytotoxic therapy from those of immunotherapy. This overlap between immunotherapy exposure and response prevents distinguishing whether the observed TIME features reflect baseline tumor biology or increased sensitivity to immunotherapy. Future studies specifically designed with treatment-homogeneous cohorts or stratified by immunotherapy exposure will be required to address this limitation and to disentangle baseline versus therapy-induced TIME features.

For these reasons, our findings should not be interpreted as predictive biomarkers of response but rather as hypothesis-generating observations. Rather than identifying baseline predictive biomarkers, our findings should be interpreted as describing TIME states observed in the context of specific treatment exposures and outcomes. The spatial features described here highlight distinct immune organizations associated with different pathological outcomes, suggesting that TIME architecture may play a role in shaping treatment sensitivity or resistance in LAR+ TNBC. However, validation in larger, independent, and treatment-homogeneous cohorts will be essential to determine their reproducibility and clinical relevance.

Importantly, the strength of this study lies in its spatially resolved approach, which enables the investigation of immune–tumor interactions that cannot be captured by bulk or single-marker analyses. By focusing on spatial relationships rather than solely on cell densities, we provide a conceptual framework for understanding how immune niches may influence tumor behavior and therapeutic response. Future studies integrating spatial proteomics, transcriptomics, and longitudinal sampling will be required to confirm these observations and to assess whether TIME architecture can be leveraged for patient stratification or for the rational design of combination therapies in LAR+ TNBC.

## Data Availability

The raw data supporting the conclusions of this article will be made available by the authors, without undue reservation.

## Supplementary material

Supplementary Figure 1 Representative single-channel fluorescence images of PANCK-PD-L1-FOXP3-CD4-CD8-CD20-PD-1-TIM3 panel in Resp patient pre-therapy.

Supplementary Figure 2 Representative single-channel fluorescence images of PANCK-PD-L1-FOXP3-CD4-CD8-CD20-PD-1-TIM3 panel in Resp patient post-therapy.

Supplementary Figure 3 Representative single-channel fluorescence images of PANCK-PD-L1-FOXP3-CD4-CD8-CD20-PD-1-TIM3 panel in NoResp patient pre-therapy.

Supplementary Figure 4 Representative single-channel fluorescence images of PANCK-PDL1-FOXP3-CD4-CD8-CD20-PD-1-TIM3 panel in NoResp patient post-therapy.
