# Supplementary figures and images for: Spatial organization of the tumor immune microenvironment in LAR+ triple-negative breast cancer
Source: Front Immunol. 2026 May 22;17:1810096. doi: 10.3389/fimmu.2026.1810096 (PMC13236650; doi:10.3389/fimmu.2026.1810096)

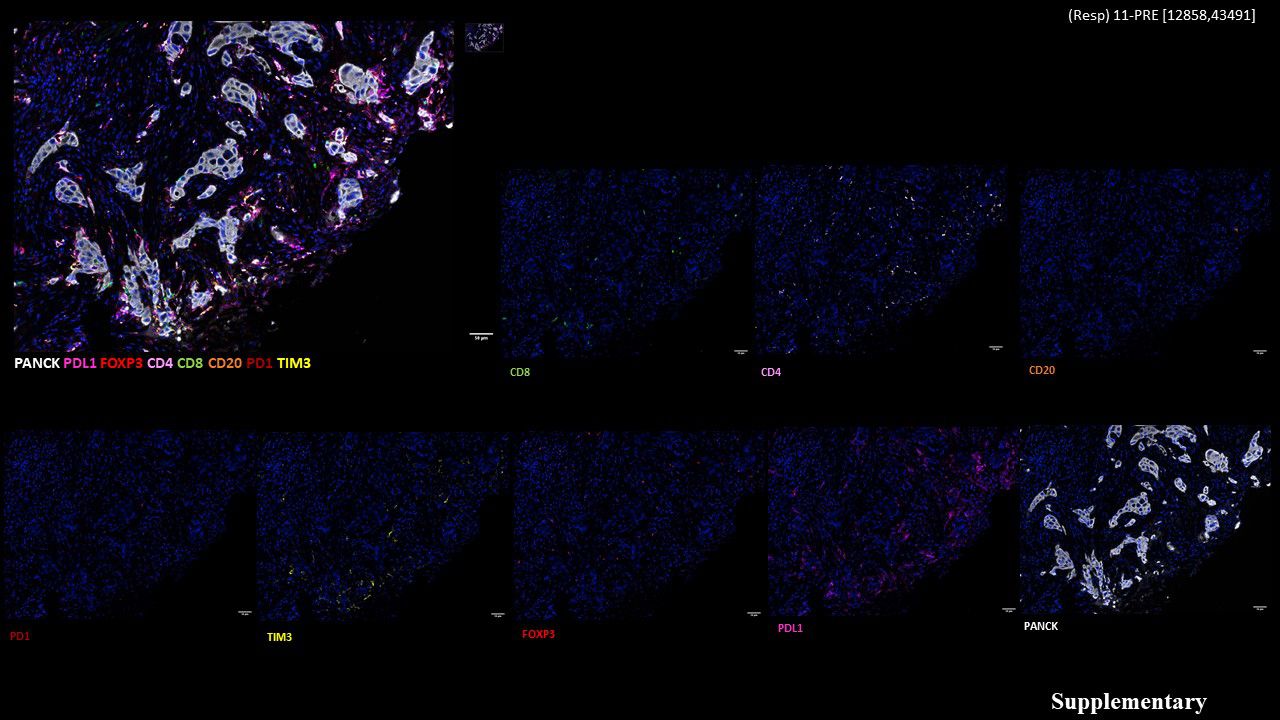

Supplement: Supplementary Figure 1 — Representative single-channel fluorescence images of PANCK-PD-L1-FOXP3-CD4-CD8-CD20-PD-1-TIM3 panel in Resp patient pre-therapy. [file Image1.jpeg]

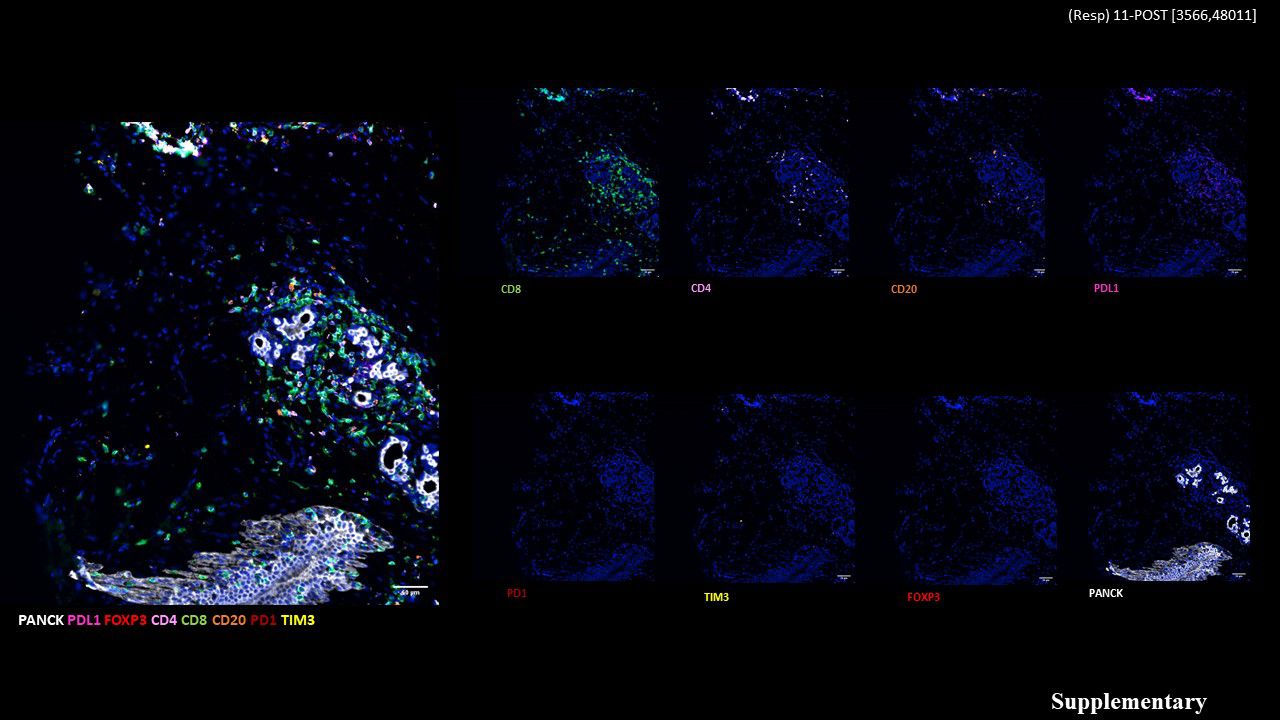

Supplement: Supplementary Figure 2 — Representative single-channel fluorescence images of PANCK-PD-L1-FOXP3-CD4-CD8-CD20-PD-1-TIM3 panel in Resp patient post-therapy. [file Image2.jpeg]

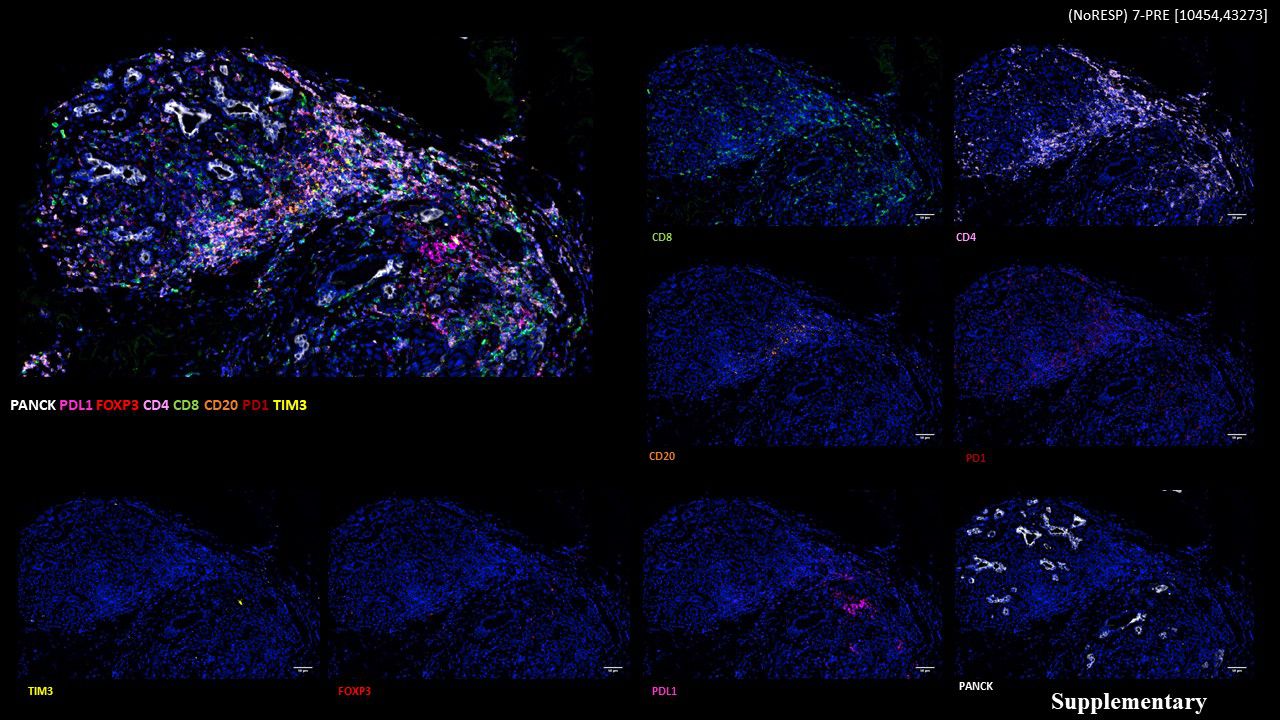

Supplement: Supplementary Figure 3 — Representative single-channel fluorescence images of PANCK-PD-L1-FOXP3-CD4-CD8-CD20-PD-1-TIM3 panel in NoResp patient pre-therapy. [file Image3.jpeg]

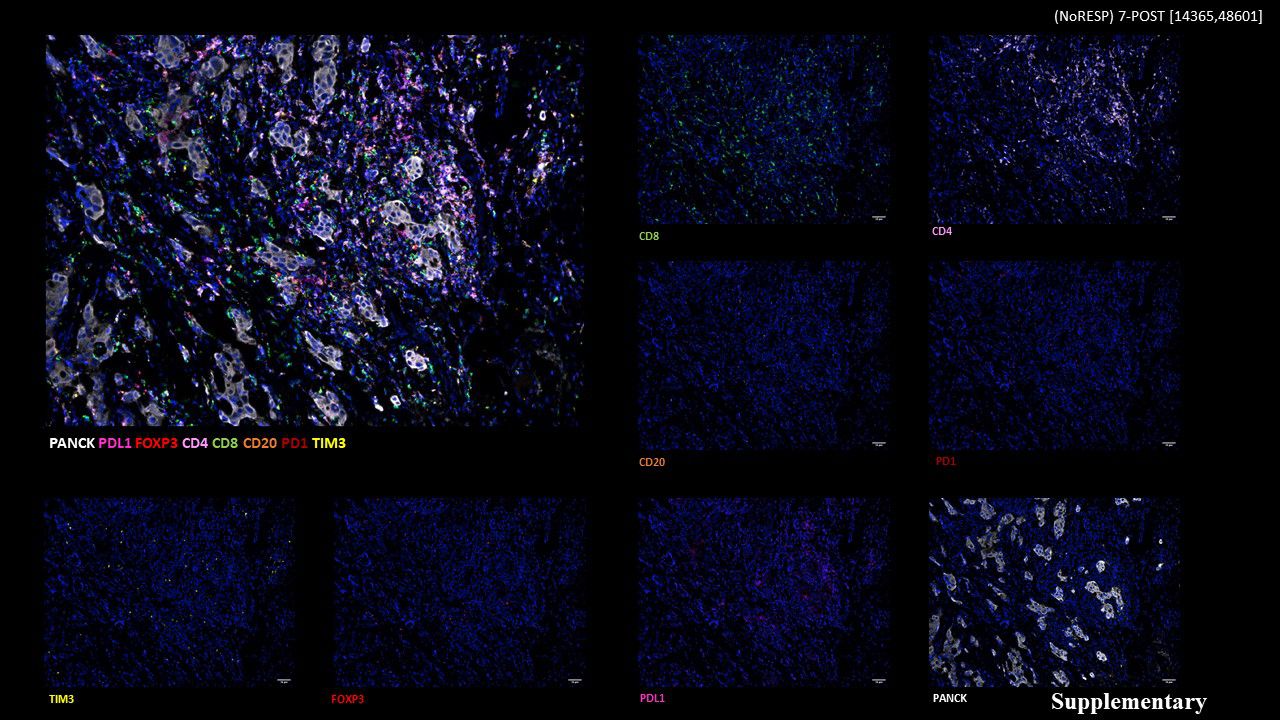

Supplement: Supplementary Figure 4 — Representative single-channel fluorescence images of PANCK-PDL1-FOXP3-CD4-CD8-CD20-PD-1-TIM3 panel in NoResp patient post-therapy. [file Image4.jpeg]
